# Primary care experiences among Brazilian adults: Cross-sectional evidence from the 2019 National Health Survey

**DOI:** 10.1371/journal.pone.0269686

**Published:** 2022-06-07

**Authors:** James Macinko, Pricila H. Mullachery

**Affiliations:** 1 Departments of Health Policy and Management and Community Health Sciences, Fielding School of Public Health, University of California, Los Angeles, California, United States of America; 2 Urban Health Collaborative, Dornsife School of Public Health, Drexel University, Philadelphia, PA, United States of America; University of South Australia, AUSTRALIA

## Abstract

**Objectives:**

The Brazilian Family Health Strategy (FHS) is strongly associated with better health system performance, but there are no nationally-representative data examining individual-level primary care experiences in the country. Here, we examine reports of primary care experiences among adults with different forms of healthcare coverage (FHS, “traditional” public health posts, and private health plans).

**Methods:**

Data are from the 2019 National Health Survey that included a shortened version of the Primary Care Assessment Tool (PCAT). PCAT questions were administered to a subsample of randomly-selected adults who had a doctor visit within the past 6 months and sought care in a primary care setting (9677 respondents). We used linear regression to examine the association between type of healthcare coverage and PCAT scores adjusted for sex, age, socioeconomic status, health status, geographic region and state of residence.

**Results:**

Primary care experiences in the sample of Brazilians who had a doctor visit 6 months prior to the survey averaged a modest PCAT score of 57 out of 100. Regression models show that users of the FHS had superior primary care experiences, but with large variations across Brazilian regions and states. Individuals selected to respond to the PCAT questions were more likely to be female, older, and poorer, and to be in worse health than the general population.

**Conclusions:**

Brazil’s FHS is associated with modest, but higher-reported primary care experiences than both traditional public health posts and those who have a private health plan. Future iterations of the PCAT module could enhance generalizability by including individuals who had a doctor visit in the past 12 (instead of 6) months.

## Introduction

Primary health care (PHC) is an essential aspect of health systems and is considered an essential strategy for achieving the Sustainable Development Goals [1]. Brazil has invested heavily in PHC, primarily through its Family Health Strategy (FHS), a community- and team-based approach to providing preventive and curative primary care that is located near to people’s homes and offers services that are free of charge at the point of delivery [2]. Evidence shows that the FHS coverage is strongly associated with reductions in infant mortality [3], mortality due to cerebrovascular and hearth diseases [4], and mortality amenable to health care [5]. Expansion of FHS coverage has also been associated with reductions in inequities in access to health care [6] and inequities in mortality by race/ethnicity [7]. However, most existing studies on PHC in Brazil are either based on ecological-level data or have been conducted in a limited number of municipalities or states. As a result, there are no nationally-representative, population-based studies of individual-level experiences of PHC in the country.

A recent initiative undertaken by the Brazilian Ministry of Health and the Brazilian Institute of Geography and Statistics (IBGE) endeavored to include a shortened version of the Primary Care Assessment Tool (PCAT), an instrument for measuring individual primary care experiences, in the country’s 2019 National Health Survey (*Pesquisa Nacional de Saúde* or PNS) [8]. The PCAT is a validated tool that assesses each aspect of primary care performance (access and first contact care, longitudinal care, care coordination, comprehensive care, family and community orientation, healthcare provider communication and cultural competence) and has been adapted and used in dozens of countries to date [9]. Previous studies using the PCAT (or close adaptations of the tool) in Brazilian cities and states have found substantial variation in primary care experiences in different geographic areas [10–13] and between users of different types of primary healthcare in the country [14]. Some studies also highlighted particularly high performance in some domains such as cultural competence and longitudinality, while generally lower performance has been found in the domains of access, family and community orientation, and comprehensiveness of care [11, 13, 15], suggesting the need to further strengthen these aspects within Brazil’s national public health service, the Unified Health System (or SUS).

In this article, we present results from analyses of the PNS 2019 survey to: 1) report the distribution of adults’ primary care experiences throughout the country; 2) assess whether different forms of healthcare coverage (the Family Health Strategy, “traditional” public health posts, and private healthcare providers) were associated with higher or lower reporting of good primary care experiences; and 3) determine the extent to which the results obtained are generalizable to the rest of the adult Brazilian population.

## Materials and methods

We used data from the 2019 edition of the PNS. The PNS uses a complex sample design in three stages. In the first stage, primary sampling units (PSU), represented by census tracts, are randomly selected from a master file of census tract stratified by geographic region, and urban/rural situation. In the second stage, households are selected from PSUs. Finally, adult respondents are randomly selected from within households for more detailed interviews. Pre-scheduled face-to-face interviews were conducted using structured questionnaires that cover a range of demographic, socioeconomic, and health-related questions [16, 17].

PCAT questions were administered to a subsample of adults in the selected households who answered that 1) they had a doctor visit within the past 6 months (53,484 retained, 35,047 excluded), 2) this was not their first visit to that doctor (26,532 retained, 26,952 excluded), and 3) they sought care in a public primary care setting (9,677 retained, 17,275 excluded). Our analytic sample included 9,677 respondents 18 years and older. Approximately 3.8% of respondents had at least one missing response and were dropped from the analyses resulting in a final sample size of 9,313. See study flowchart in S1 Fig.

We followed an analytic strategy consistent with previous publications [18]. This entailed first dichotomizing all likert-type scales so that the responses “Definitely/probably” were coded as 1 and “Probably not/definitely not” were coded as 0. To aid interpretation, we then summed up the resulting measures, divided that sum by the number of questions, and multiplied by 100 to obtain a total PCAT score that ranged from 0 to 100. Three of the 22 PCAT questions were asked only to those who had a previous specialist visit, so for those individuals, the score’s denominator was 22, while for the remaining respondents it was 19. While the full adult PCAT scale can be broken down into its subcomponent (domain) scores, the reduced version included in the PNS-2019 had only a few indicators for each domain. While we present mean values for each PCAT question and for some domain-specific sub-scores, we do not formally analyze sub-scores, given the lack of psychometric evidence for their ability to capture primary subdimensions (e.g. access, longitudinality) in this shortened instrument.

The first set of analyses were intended to identify how Brazil’s three main types of primary healthcare (Family Health Strategy, traditional public health clinics, and private health plans) differed with respect to mean PCAT scores after controlling for a range of demographic, socioeconomic, health, healthcare, and geographic factors likely to differ across users of these different types of services. We note that although respondents were selected based on whether they had consulted in the public sector, a proportion of these individuals also had a private health plan, so we assess these individuals as their own subgroup.

The second set of analyses assessed the relationship between socio-demographic, health and other characteristics and whether the respondent was selected for the PCAT questions. In both analyses, we controlled for factors that might confound the relationship between healthcare coverage and primary care experiences, including respondent age (continuous), birth sex (male, female), education (categorized as less than primary school, primary complete, high school complete, and any college or more), household wealth (derived from principal components analysis of a list of household goods and categorized into quintiles), smoking status (ever smoker versus never), and geography (either Brazilian macro-region or dummy variables for 27 jurisdictions, representing Brazil’s 26 states and the Federal District). Health status was measured by poor/very poor self-rated health (versus very good/good/fair) and self-reported previous diagnosis of seven chronic conditions or risk factors (hypertension, arthritis, asthma, cancer, depression, diabetes, and heart disease). We then constructed two variables, “any chronic condition”, defined as any of the conditions listed previously, and “multimorbidity”, defined as reporting two or more conditions.

### Statistical analysis

We first describe weighted proportions/means and calculated 95% confidence intervals for all variables and the mean PCAT scores for each category. Tests of statistical significance were design-corrected Wald or F-tests, due to the complex survey design and sampling weights. We then performed ordinary least squares (OLS) regression to assess factors associated with PCAT scores, while controlling for the covariates described above. We tested three models: (1) unadjusted ones assessing the bivariate association between type of healthcare coverage (i.e. FHS, traditional, or private) and PCAT score; (2) a model with adjustments for all individual-level covariates and the 5 Brazilian geographic regions; and (3) a model similar to model 2 but instead of adjusting for regions we included dummy variables for 27 jurisdictions (26 states and federal district). We then calculated marginal effects for the 27 jurisdictions stratified by the type of healthcare coverage to produce the mean adjusted value of the PCAT score by state and healthcare coverage and then plotted the results. Finally, we performed logistic regression analyses to assess the relationship between those who were and who were not selected to answer questions on their primary care experiences and estimated the adjusted odds ratios for different characteristics associated with being selected to respond to the PCAT question. This was done to elucidate any factors that may differ between the two populations.

We used STATA 17 for all analytical procedures. All analyses accounted for complex survey design and individual sampling weights.

This study used publicly-available and de-identified version of the PNS 2019 survey and was therefore considered exempt from further human subjects review. The Brazilian National Research Ethics Commission approved the PNS-2019 in August 2019 (No 3529376). The datasets analyzed in the current study are freely available from the Brazilian Institute of Geography and Statistics (IBGE) at the following URL: https://www.ibge.gov.br/en/statistics/social/health/16840-national-survey-of-health.html?=&t=o-que-e

## Results

Items from the PCAT scale, the primary care domain they represent, the weighted proportion of positive responses, and Cronbach’s alpha values are presented in Table 1. Overall, the average PCAT score was 57 out of 100, indicating a moderate score across the sample of respondents. There was significant variation in terms of scores associated with different items, ranging from a high of 83.25% responding that they could easily share problems with their doctor to a low of 35.66% reporting it was easy to get health advice over the phone. Mean primary care sub-domain scores also differed. The lowest scored domain was access (55) while the highest were coordination (71) and longitudinality (71). The scale combining all items had high internal validity (Cronbach’s alpha of 0.8536) with no suggestion that any item should be removed from the score (**Table** 1).

**Table 1:** Specific PCAT items, corresponding primary care domain, and Cronbach alpha scores.

PCAT item	Primary care domain	Weighted proportion responding affirmatively	Cronbach’s Alpha^a^
Doctor is first contact for new/ongoing problems	First contact/ Access	75.06	0.8506
Easy to get advice by telephone	Access	35.66	0.8516
Not difficult to obtain care	Access	54.13	0.8602
**Access items mean score**		**54.95**	**-**
Seen primarily by the same doctor	Longitudinality	71.72	0.8518
Can easily share problems with doctor	Longitudinality	83.25	0.849
Doctor best knows your problems	Longitudinality	73.56	0.8443
Would not want to change doctor	Longitudinality	54.39	0.8534
**Longitudinality items mean score**		**70.73**	-
Doctor recommended a specialist	Coordination	80.39	0.8490
Doctor gives written information for referral	Coordination	66.17	0.849
Doctors knows results of specialist visit	Coordination	70.59	0.8474
Doctor interested in specialist visit	Coordination	72.57	0.8466
You can view your medical charts	Coordination	66.24	0.8513
**Coordination items mean score**		**71.19**	**-**
Your doctor would provide the following:			-
-mental health counseling	Comprehensiveness	60.94	0.8444
-smoking cessation advice	Comprehensiveness	58.60	0.8462
-advice about aging	Comprehensiveness	61.99	0.8424
-advice about diet	Comprehensiveness	77.60	0.8436
-advice about exercise	Comprehensiveness	73.81	0.845
-advice on preventing falls	Comprehensiveness	56.15	0.8427
**Comprehensiveness items mean score**		**64.85**	**-**
Doctor verifies/discusses your medications	Communication	76.26	0.8444
Doctor asks about your ideas	Communication/ Cultural competence	46.75	0.8431
**Communication items mean score**		**61.50**	**-**
Doctor would meet with your family	Family orientation	56.18	0.8443
Doctor conducts patient surveys	Community orientation	44.21	0.848
**Overall test scale (all items)**		**57.06**	**0.8536**

^a^Alpha for each item indicates the overall Cronbach’s alpha score if the item were to be deleted from the scale.

Among PCAT respondents, approximately 70% were female and the average age was 49 years. Respondents were more likely to be in the lowest quintiles of the household asset index (i.e., poorer) and about 52% had not completed primary school. Approximately half of the PCAT respondents reported having fair or poor health, 60% reported at least one of seven chronic conditions, and 27% reported 2 or more chronic conditions. Regarding the type of healthcare coverage, 72% of respondents reported their household was registered with the FHS, 5% reported having a private health plan and the remaining 21% were assumed to be covered by traditional public health posts. Regarding region of residence, 41% of the respondents were from the Southeast, 28% from the Northeast, 17% from the South, and 6% from both North and Midwest regions. Finally, 18% of the respondents resided in a rural area **(****Table** 2**)**.

**Table 2:** Distribution of respondent characteristics, mean PCAT score and bivariate analyses, PNS 2019.

	PCAT responders Weighted % 95% CI		PCAT score Mean 95% CI		Coef^a^	95% CI
Traditional public healthcare	21.94	[20.15,23.86]	51	[49,54]	Ref	-
Family Health Strategy (FHS)	72.45	[70.43,74.38]	58	[57,59]	6.77***	[4.67,8.87]
Private health plan	5.61	[4.73,6.64]	58	[54,62]	6.6**	[1.98,11.21]
Male	30.15	[28.55,31.79]	58	[56, 59]	Ref	-
Female	69.85	[68.24,71.41]	57	[55, 58]	-1.27	[-2.92,0.39]
Age (mean) in years	49.23	[48.59,49.86]	n/a		0.15***	[0.10,0.19]
Goods quintile 1 (poorest)	26.77	[25.41,28.18]	57	[55, 58]	Ref	-
Quintile 2	25.03	[23.68,26.42]	58	[56, 59]	1.1	[-0.80,3.00]
Quintile 3	21.79	[20.37,23.29]	57	[56, 59]	0.75	[-1.52,3.01]
Quintile 4	16.92	[15.47,18.48]	56	[53, 58]	-0.78	[-3.63,2.07]
Quintile 5 (richest)	9.49	[8.31,10.82]	57	[54, 61]	0.43	[-3.19,4.05]
Less than primary school	51.82	[50.03,53.60]	58	[57, 60]	Ref	-
Primary school complete	14.47	[13.32,15.69]	56	[54, 58]	-2.4*	[-4.62,-0.18]
High School complete	24.6	[23.07,26.21]	56	[54, 58]	-2.39*	[-4.27,-0.50]
Some college or more	9.11	[8.04,10.32]	54	[51, 57]	-4.12*	[-7.45,-0.79]
Never smoker	87.58	[86.37, 88.70]	57	[56, 58]	Ref	-
Ever smoker	12.42	[11.31,13.62]	57	[55, 60]	0.43	[-2.21,3.07]
Very/good self-rated health	50.47	[48.63, 52.31]	57	[55, 58]	Ref	-
Poor self-rated health	49.53	[47.71,51.34]	58	[57, 59]	1.07	[-0.63,2.76]
No chronic conditions	39.92	[38.33,41.53]	53	[52, 55]	Ref	-
Any chronic condition	60.08	[58.46,61.69]	59	[58, 60]	5.87***	[4.16,7.57]
Two+ chronic conditions	27.76	[26.22,29.36]	61	[60, 63]	5.86***	[4.15,7.56]
North region	6.79	[6.21,7.42]	54	[51, 56]	Ref	-
Northeast region	28.21	[26.69,29.79]	56	[54, 57]	2.21	[-0.22,4.64]
Southeast region	41.12	[39.04,43.22]	56	[55, 58]	2.88*	[0.24,5.53]
South region	17.74	[16.39,19.16]	62	[60, 64]	8.61***	[5.76,11.46]
Mid-west region	6.14	[5.54,6.81]	57	[55, 59]	3.47*	[0.35,6.58]
Urban residence	81.95	[80.87,82.98]	57	[56, 58]	Ref	-
Rural residence	18.05	[16.78,19.40]	59	[57, 60]	1.90*	[0.01,3.78]
Total	n/a		57	[56, 58]	n/a	

Numbers are weighted sample proportions, mean PCAT scores and 95% confidence intervals for each respondent characteristic.

^a^Regression coefficient and 95% confidence intervals from bivariate linear regression analysis of each independent variable with the PCAT score.

*p<0.05

**p<0.01

***p<0.001

Table 2 shows that individuals whose household was covered by the FHS and those who had a private health plan reported higher mean PCAT scores than users of traditional public health clinics (see histograms for PCAT score distribution by type of healthcare coverage in S2 Fig). In terms of demographic and socioeconomic groups, there were no significant differences in mean PCAT scores by sex or household assets, while those with less than primary school reported slightly higher mean PCAT scores than those with higher educational attainment. In terms of health status, smokers and never-smokers had similar mean PCAT scores, and those with good/very good self-rated health had similar mean PCAT scores as those with poor health. People with any chronic condition and those with multimorbidity had higher PCAT scores than those with no chronic conditions. In terms of geographic distribution, residents in the South, Mid-west, and Southeast regions had higher mean PCAT scores than residents in the North (reference) region, and those in rural areas had slightly higher mean PCAT scores than those in urban areas. **(****Table** 2**).**

In regression analyses, PCAT scores differed considerably by type of healthcare coverage. Users of the FHS had on average 6.76 (unadjusted- model 1) and 6.2 (adjusted–model 2) points higher PCAT score than users of traditional public health clinics. In the third model, including state fixed effects, users of the FHS had a 5.5 point higher score than users of traditional health clinics **(****Table** 3**)**. Older respondents, those with any chronic condition, and those with multimorbidity had higher PCAT scores, on average, in comparison with their younger and healthier counterparts. Individuals reporting poor self-rated health had lower PCAT scores that those with good/very good self-rated health, after adjusting for covariates. Finally, respondents in the South had higher PCAT scores, as compared to the reference region (North) **(****Table** 3**).**

**Table 3:** Regression analyses of the association between respondents’ individual characteristics and their primary care experiences (PCAT score).

	Model 1	Model 2	Model 3
Traditional public clinics	Ref	Ref	Ref
(UBS)			
FHS coverage	6.76***	6.2***	5.55***
	4.67,8.87	4.11,8.29	3.37,7.72
Private health plan	6.59**	3.93	3.35
	1.98,11.21	-0.69,8.55	-1.22,7.91
Male		Ref	Ref
Female		-1.53	-1.46
		-3.25,0.18	-3.15,0.24
Age (continuous)		0.09**	0.09**
		0.03,0.15	0.03,0.15
Household goods Q1		Ref	Ref
(poorest)			
Household goods Q2		0.64	0.48
		-1.31,2.58	-1.46,2.41
Household goods Q3		0.31	0.37
		-2.10,2.73	-2.00,2.74
Household goods Q4		-1.2	-1.01
		-4.32,1.91	-4.08,2.07
Household goods Q5		-0.38	-0.35
(richest)		-4.26,3.50	-4.23,3.52
Less than primary school		Ref	Ref
Primary school complete		-0.42	-0.38
		-2.86,2.02	-2.79,2.02
High school complete		0.58	0.85
		-1.63,2.79	-1.35,3.06
Some college and more		-0.89	-0.52
		-4.78,3.00	-4.40,3.36
Never smoker		Ref	Ref
Ever smoker		-0.1	0.05
		-2.69,2.49	-2.49,2.60
Very good/good self-		Ref	Ref
rated health			
Poor self-rated health		-2.1*	-1.93*
		-4.01,-0.20	-3.81,-0.04
No chronic conditions		Ref	Ref
Any chronic condition		3.32**	3.4**
		1.12,5.52	1.21,5.59
2+ chronic conditions		3.45**	3.51***
		1.39,5.51	1.46,5.56
Urban		Ref	Ref
Rural		1.86	1.8
		-0.13,3.86	-0.18,3.79
North region		Ref	Ref
Northeast region		0.97	
		-1.41,3.34	
Southeast		1.89	
		-0.70,4.48	
South		6.29***	
		3.38,9.20	
Midwest		2.04	
		-1.05,5.13	
States included	No	No	Yes
Adjusted R^2^	0.0155	0.0540	0.0664
N	9,313	9,313	9,313

Results are beta coefficients and their 95% confidence intervals from survey weighted linear regression that controls for all variables within each column.

*p<0.05

**p<0.01

***p<0.001

**Fig 1:**
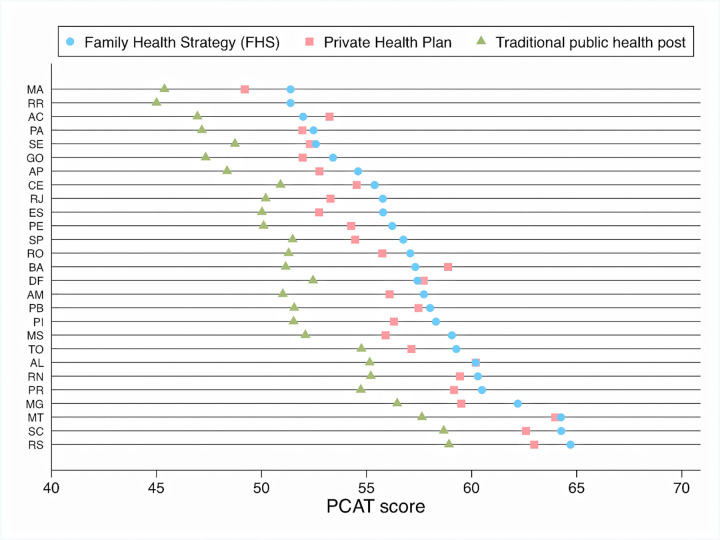
Adjusted PCAT scores, by main type of healthcare coverage and Brazilian state. Numbers are mean PCAT scores (from 0 to 100), adjusted for all variables in model 3, Table 2 and stratified by type of healthcare coverage. MA (Maranhão), RR (Roraima), AC (Acre), PA (Pará), SE (Sergipe), GO (Goiás), AP (Amapá), CE (Ceará), RJ (Rio de Janeiro), ES (Espírito Santo), PE (Pernambuco), SP (São Paulo), RO (Rondônia), BA (Bahia), DF (Distrito Federal), AM (Amazonas), PB (Paraíba), PI (Piauí), MS (Mato Grosso do Sul), TO (Tocantins), AL (Alagoas), RN (Rio Grande do Norte), PR (Paraná), MG (Minas Gerais), MT (Mato Grosso), SC (Santa Catarina), RS (Rio Grande do Sul). *Insufficient sample size to calculate score for private health plan holders in Roraima (RR).

There were large variations in user experiences across states with mean PCAT scores (adjusted for all individual-level covariates) ranging from 65 for FHS users in Rio Grande do Sul (RS) to 45 among users of traditional health clinics in Maranhão (MA), one of the poorest states in Brazil. FHS users had higher scores than users of traditional public health clinics in all states. Individuals with private health plans had higher scores compared to users of traditional public health clinics; in some states individuals with private plans had slightly higher scores than those reported by FHS users **(Fig 1).**

Table 4 shows that individuals selected for the PCAT questions were different from the general population in several important dimensions. PCAT respondents were more likely to be female, older, poorer (having fewer household goods), have lower educational attainment, were more likely to be covered by the FHS, and were less likely to have a private health plan, after adjusting for covariates. In terms of health status, individuals selected for the PCAT questions (vs. those who were not) are more likely to have poor self-rated health and more likely to have any chronic condition or multimorbidity. There was no difference in smoking status. PCAT respondents were also more likely to live in the Southeast and South regions, but there was no significant difference between rural and urban residents **(****Table** 4**).**

**Table 4:** Comparison of characteristics of respondents selected and not selected for PCAT questions.

	Weighted %		Adjusted OR (PCAT/No PCAT
	PCAT N = 9,677	No PCAT N = 78,854	
Traditional public healthcare coverage	21.94	23.56	Ref
	[20.15,23.86]	[22.74,24.39]	
Family Health Strategy (FHS)	72.45	46.82	1.57***
	[70.43,74.38]	[45.86,47.78]	1.42,1.74
Private health plan	5.61	29.63	0.21***
	[4.73,6.64]	[28.80,30.46]	0.17,0.27
Male	30.15	48.87	Ref
	[28.59, 31.76]	[48.23,49.51]	
Female	69.85	51.13	2.13***
	[68.24,71.41]	[50.49,51.77]	1.95,2.32
Age (mean)	49.23	44.41	1.01**
	48.59,49.86	44.15,44.66	1.00,1.01
Household goods Quintile 1 (poorest)	26.77	19.17	Ref
	[25.41,28.18]	[18.65,19.70]	
Quintile 2	25.03	19.39	0.98
	[23.68,26.42]	[18.85,19.93]	0.89,1.08
Quintile 3	21.79	19.8	0.96
	[20.37,23.29]	[19.19,20.41]	0.86,1.08
Quintile 4	16.92	20.36	0.87*
	[15.47,18.48]	[19.78,20.94]	0.75,1.00
Quintile 5 (richest)	9.49	21.29	0.71***
	[8.31,10.82]	[20.50,22.11]	0.59,0.84
Less than primary school	51.82	32.68	Ref
	[50.03,53.60]	[31.99,33.37]	
Primary school complete	14.47	14.48	0.86*
	[13.32,15.69]	[14.04,14.94]	0.76,0.97
High School complete	24.6	30.45	0.85**
	[23.07,26.21]	[29.81,31.09]	0.75,0.95
Some college or more	9.11	22.39	0.7***
	[8.04,10.32]	[21.64,23.16]	0.59,0.84
Never smoker	87.39	87.58	Ref
	[86.96,87.82]	[86.38,88.69]	
Ever smoker	12.42	12.61	0.9
	[11.31,13.62]	[12.18,13.04]	0.80,1.02
Very/good self-rated health	50.47	69.55	Ref
	[48.66,52.29]	[68.94,70.15]	
Poor self-rated health	49.53	30.45	1.25***
	[47.71,51.34]	[29.85,31.06]	1.14,1.37
No chronic condition	39.92	60.44	Ref
	[38.31,41.54]	[59.79,61.09]	
Any chronic condition	60.08	39.56	1.67***
	[58.46,61.69]	[38.91,40.21]	1.52,1.84
Two or more chronic conditions	27.76	14.67	1.24***
	[26.22,29.36]	[14.22,15.14]	1.11,1.38
North region	6.79	7.98	Ref
	[6.21,7.42]	[7.70,8.27]	
Northeast region	28.21	26.24	1.11
	[26.69,29.79]	[25.67,26.82]	1.00,1.25
Southeast region	41.12	43.73	1.43***
	[39.04,43.22]	[42.89,44.57]	1.26,1.63
South region	17.74	14.31	1.75***
	[16.39,19.16]	[13.88,14.76]	1.52,2.01
Mid-west region	6.14	7.74	1.09
	[5.54,6.81]	[7.44,8.05]	0.93,1.27
Urban residence	81.95	86.69	Ref
	[80.6, 83.22]	[86.29, 87.08]	
Rural residence	18.05	13.31	1.06
	[16.78,19.40]	[12.92,13.71]	0.95,1.19

Adjusted Odds Ratios calculated using survey-adjusted weighted logistic regression that controls for all other variables listed in table.

*p<0.05

**p<0.01

***p<0.001

## Discussion

Primary care experience, as measured by a reduced version of the Primary Care Assessment Tool in a sample of adult Brazilians who had a doctor visit in a public primary care setting 6 months prior to the survey, was slightly above average, with a mean PCAT score of 57 out of 100. Individuals whose households were covered by the FHS had consistently higher PCAT scores than those served by traditional public health clinics and those with private health plans who recently used public healthcare services, once individual-level factors were statistically controlled. Adjusted models showed that older respondents, those with any chronic condition, and those with multimorbidity had higher PCAT scores compared to their younger and healthier counterparts, but respondents with poor (vs. good/very good) self-rated health had lower PCAT scores. We also found large variations in user experience across regions and states.

These results are largely consistent with previous studies assessing adult primary care experiences in Brazil. First, as in this study, other reports indicate that the overall PCAT score in Brazil varied across localities [10–13, 19–22]—from a 5 out 10 in Ilheus, Bahia in 2010 [10] to 6.4 out of 10 in Florianopolis, Santa Catarina in 2012 [22]–and in both cases adult user experiences were considered to be less than adequate. Second, studies that compared primary care experiences of FHS users with those of traditional public health clinics, found that the former reported higher PCAT scores [10, 12, 13]. Third, among specific primary care domains, access was consistently a low scoring component [10, 12, 13, 19, 21, 22] while longitudinality was one of the highest scoring components [10, 12, 13, 19]. Access-related issues such as poor access to care for acute conditions and unscheduled visits, poor access to information via phone or other non-face-to-face channels, and long waiting time have also been reported by other studies [20, 21]. On the other hand, the FHS model which provides care near people’s homes most likely leads to better care continuity. This is consistent with higher longitudinality scores for FHS users compared to users of traditional health clinics as reported by other studies [10, 12, 13].

Coordination of care, also a top scoring component in our study, was more likely to vary across the PCAT literature in Brazil [12, 13, 22]. Coordination of care is also less consistent when we compare responses from individual adult healthcare users with those of healthcare providers, with the latter usually assessing this dimension more highly than users [13, 14, 19, 20]. Coordination of care is likely to vary due to barriers to specialist services, however, results of the current (2019) survey show that coordination received a relatively high score, which could point to improvements in this component in recent years.

Despite the extensive literature on adult primary care experiences in Brazil, particularly using the PCAT, individual-level predictors of primary care experiences have been examined infrequently [15]. In addition, studies comparing users of FHS and traditional public health clinics have not always adjusted for individual-level characteristics [12, 13, 19, 22] which prevents such analyses from ruling out potential biases due to differences in the composition of these two groups. Our study measured PCAT scores across different users and found that once socioeconomic, demographic, and health-related factors are statistically accounted for, users of the FHS had higher PCAT scores than users of traditional health clinics. From an equity perspective, our results suggest that among those covered by the FHS, those from lower socioeconomic backgrounds reported experiences similar to those from higher socioeconomic background (based on household items and educational attainment). On the other hand, state-specific scores revealed large variation in user primary care experiences, which is a concerning finding, but consistent with the studies cited above. This observation may provide support to policies that invest in strengthening FHS teams in locations where PCAT scores are particularly low. The relationship between lower PCAT scores and regions that have traditionally faced physician shortages would also lend support to reconstituting the well-evaluated *Mais Médicos* program that had successfully expanded primary care doctor availability to remote and hard to reach areas of the country before being abruptly terminated [23].

The PNS 2019 did not apply the PCAT questions to all respondents. Instead, it selected people based on their most recent doctor visit (less than 6 months), whether they had seen that doctor before, and whether they had sought care in a primary care setting within the national health service. Some of these inclusion criteria are present in the original PCAT tool and were designed to assure that respondents were able to answer questions about having received ongoing care from a particular healthcare provider. However, given that longitudinal care is a feature of good primary care, selecting respondents based on whether they had previously gone to the same doctor may have artificially inflated PCAT scores. Moreover, selecting individuals based on having sought care within the past 6 months may have resulted in a sample of respondents who are in worse health, have a higher propensity to seek healthcare, or who may have fewer barriers to care than those who did not seek care within this relatively short time period. Evidence for this selection bias is clear when comparing rates of hypertension (41.7% among the less than 10,000 PCAT respondents, versus only 25.9 among the over 86,000 respondents for the entire survey, data not shown). Higher reporting of chronic conditions in the group selected to respond to the PCAT questions could be partially due to better detection of these conditions in this group due to fewer barriers to access. Based on the results reported here, while it was possible to capture primary care experiences across a board range of respondents, the extent to which these results can be generalized to the entire adult Brazilian population is limited.

This study has several important limitations. First, all data are based on self-report and are thus subject to both recall and social desirability biases. Second, as discussed above, given the way the PCAT was administered in the PNS 2019, results from this study cannot be used to draw conclusions about the user experience of the entire Brazilian adult population, but only for those who have relatively high accessibility to health care within the national health service. Future iterations of the PNS/PCAT module could enhance generalizability by including individuals who had a doctor visit in the past 12 (instead of 6) months. This would effectively double the sample size and potentially include a sample of individuals more similar to the general population in terms of their demographic, socioeconomic, and health-related characteristics. Third, we categorized respondents based on the type of overall healthcare coverage they reported in the survey. That is, we assumed that people with a private health plan had previous experiences with doctors working in that health plan and that people covered by the FHS were referring to doctors working in the FHS, but it is possible people reported experiences based on a different doctor or model of care. Future iterations of the PCAT tool would benefit from not restricting PCAT responses only to those who sought care in a public primary care setting, instead asking respondents to identify characteristics of the healthcare provider they are referring to when answering questions, such as whether the provider was a physician (specialist or general practitioner) or a nurse and whether they worked in the private sector, the FHS, or a traditional public clinic. Such minor modifications could greatly enhance the study’s ability to assess the entire Brazilian healthcare system.

In conclusion, this study adds to the body of research demonstrating that the FHS continues to serve as a leading example of how to scale up highly-effective primary health care in a middle-income country context. Nevertheless, the overall level of the primary care experience reported here was less than optimal. While there is no previous baseline assessment with which to compare results, it is not unreasonable to question whether the average rating of 57/100 may be in part due to changes implemented in the past years that reduced government spending on the public health system and eliminated programs designed to enhance accessibility to primary health care [24, 25]. It is strongly recommended that the next version of the PNS include the PCAT tool to be able to assess progress made and identify any new challenges that have arisen since this baseline survey. Other countries in the region could consider deploying the shortened version of the PCAT in national health surveys or developing modules to be included in ongoing data collection efforts such as the Demographic and Health Surveys (DHS). Such an approach could help to bridge the gap in monitoring and evaluating progress in achieving universal primary healthcare coverage.

## Supporting information

S1 Fig Flowchart describing PCAT question inclusion criteria and construction of final sample. (TIF)

S2 Fig Distribution of PCAT scores, by type of coverage. (TIF)
