# Supplementary figures and images for: Primary care experiences among Brazilian adults: Cross-sectional evidence from the 2019 National Health Survey
Source: PLoS One. 2022 Jun 7;17(6):e0269686. doi: 10.1371/journal.pone.0269686 (PMC9173631; doi:10.1371/journal.pone.0269686)

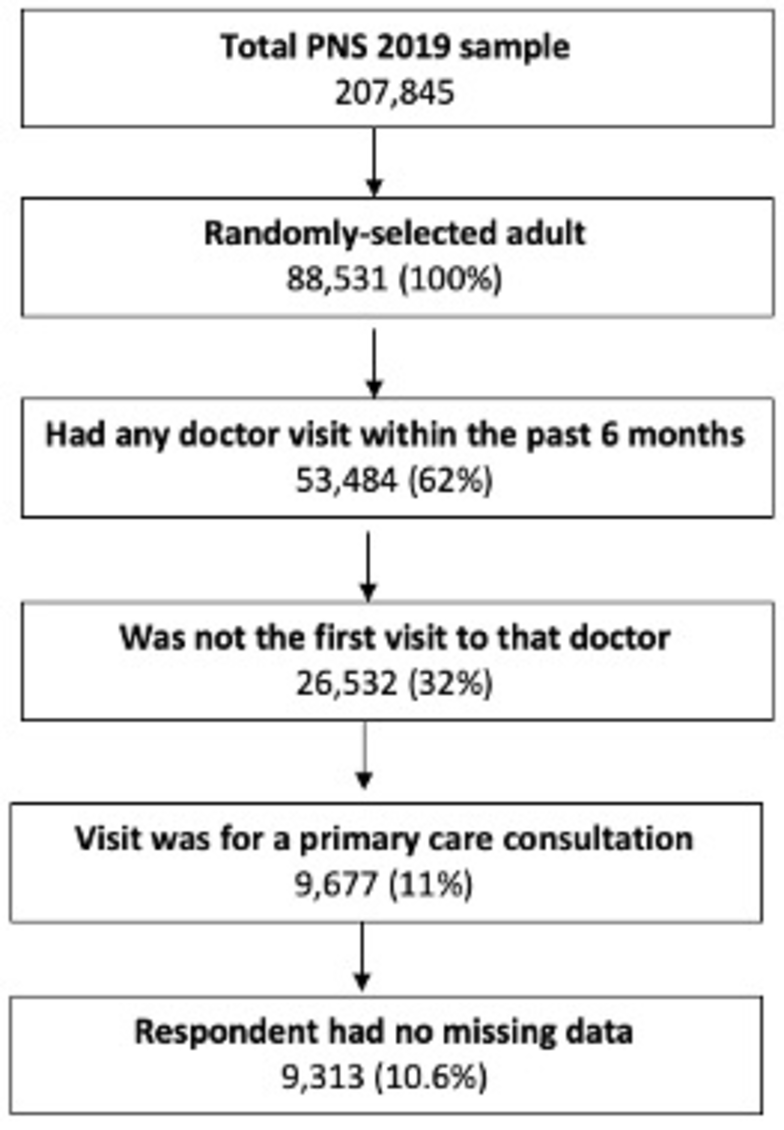

Supplement: S1 Fig — (TIF) [file pone.0269686.s001.tif]

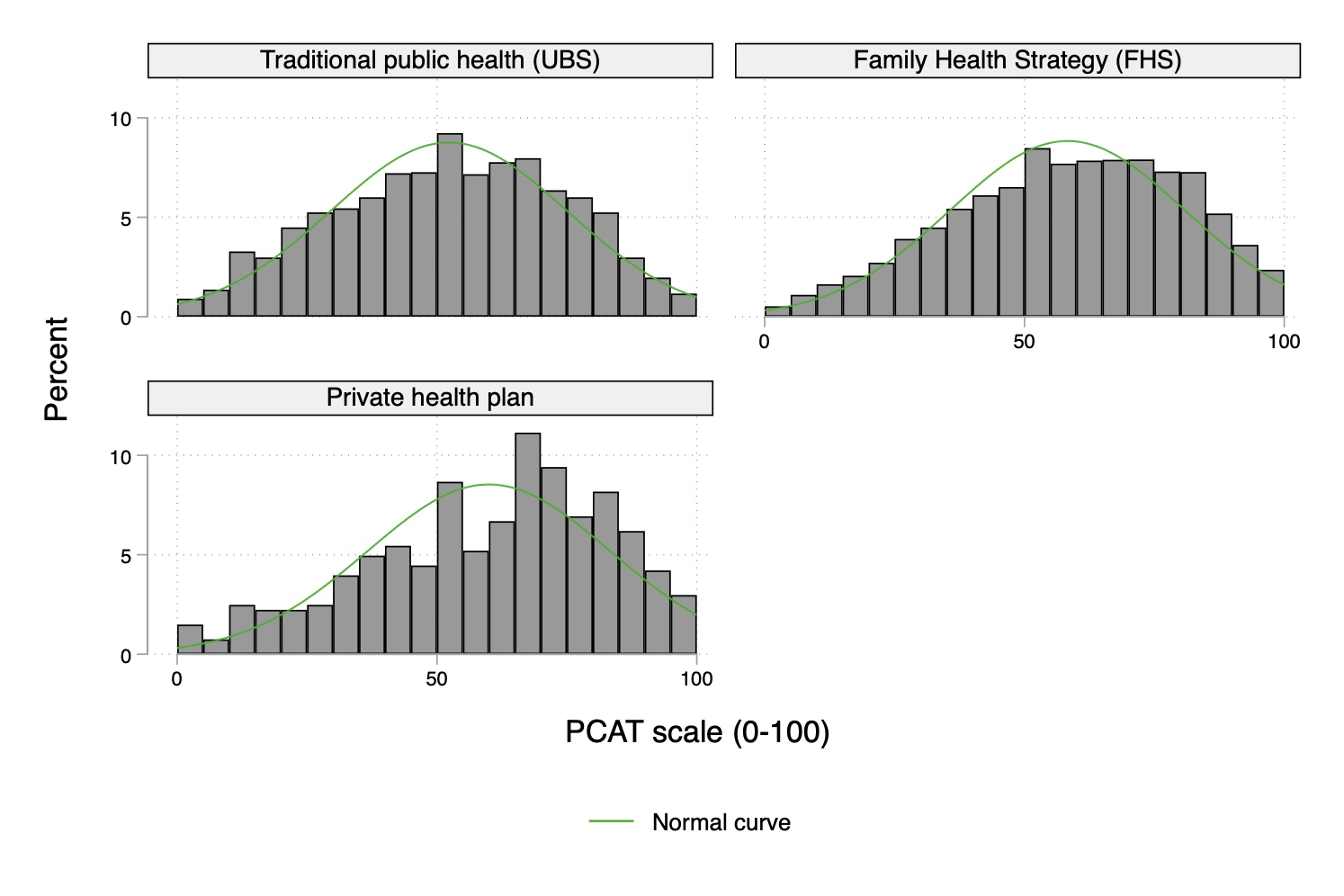

Supplement: S2 Fig — (TIF) [file pone.0269686.s002.tif]
